# Association study and expression analysis of *CYP4A11* gene copy number variation in Chinese cattle

**DOI:** 10.1038/srep46599

**Published:** 2017-05-11

**Authors:** Mingjuan Yang, Jingqiao Lv, Liangzhi Zhang, Mingxun Li, Yang Zhou, Xianyong Lan, Chuzhao Lei, Hong Chen

**Affiliations:** 1 College of Animal Science and Technology, Northwest A&F University, Shaanxi Key Laboratory of Molecular Biology for Agriculture, Yangling, Shaanxi, 712100, China; 2 Department of Forensic Medicine, Xinxiang Medical University, Xinxiang, Henan, 453003, China; 3 Library, Xinxiang Medical University, Xinxiang, Henan, 453003, China

## Abstract

The identification of copy number variations (CNVs) allow us to explore genomic polymorphisms. In recent years, significant progress in understanding CNVs has been made in studies of human and animals, however, association and expression studies of CNVs are still in the early stage. It was previously reported that the Cytochrome P-450 4A11 (*CYP4A11*) gene is located within a copy number variable region (CNVR) that encompasses quantitative trait loci (QTLs) for economic traits like meat quality and milk production. So, this study was performed to determine the presence of *CYP4A11* CNV in six distinct cattle breeds, identify its relationship with growth, and explore the biological effects of gene expression. For three *CYP4A11* CNV types, Normal was more frequent than Gain or Loss. Association analysis revealed a positive effect of *CYP4A11* copy number on growth traits (*P* < 0.05). One-way analysis of variance (ANOVA) analysis revealed that more *CYP4A11* copies increased the gene expression level. Moreover, overexpression of *CYP4A11 in vitro* revealed its effect on lipid deposit. The data provide evidence for the functional role of *CYP4A11* CNV and provide the basis for future applications in cattle breeding.

After publication of two cattle genome maps (Btau_4 and UMD3)^1^,^2^, single nucleotide polymorphisms (SNPs) were identified as a key factor causing genetic mutation in cattle, and abundant SNP information has been collected^3^,^4^,^5^. Simultaneously, many SNPs were reported to be associated with milk yield, milk protein percentage and milk fat percentage in *Bos taurus* dairy cattle^6^, and average daily gain, net feed intake, residual food intake and intramuscular fat percentage in *Bos taurus* beef cattle^6^,^7^. In addition, the distribution of quantitative trait loci (QTLs) were revealed, and artificial selection studies have been performed in dairy cattle to explore effects of genetic changes^8^,^9^. However, most SNP variants have only small effects on phenotypes, making it a priority to explain the missing heritability.

Two studies revealed that copy number variations (CNVs) that represent a significant source of genetic variation are widespread in human genomes^10^,^11^. Later, CNVs were redefined as gains or losses (insertions or deletions) larger than 50 bp in genomic sequence between two individuals of a species^12^. Soon, progress in understanding CNVs were made in human^12^,^13^, mice^14^,^15^, pig^16^,^17^, horse^18^,^19^, cattle^20^,^21^, goat^22^ and chicken^23^, using different methods including whole genome sequencing, array-based comparative genomic hybridization (aCGH), and SNP array. However, despite the significant number of investigations identifying copy number variable regions (CNVRs), few have correlated this information with association and expression analysis.

Although individual SNPs may occur more frequently, CNVs are of larger size, and thus occupy a higher percentage of genomic sequence and have potentially larger effects on phenotypic variation, including changing gene structure and dosage, altering gene regulation, and exposing recessive alleles^24^. For example, changes in the copy number of cis-regulatory elements, particularly those related to developmental genes, have been shown to influence phenotype^25^. In some cases, deleting the normal allele in a heterozygous background led to exposure of a recessive allele that led to disease^26^. CNVs that include dosage-sensitive genes can alter gene expression levels resulting in clinical phenotypes. For example, in human, *PMP22* is located within the 1.4-Mb CMT1A (Charcot- Marie -Tooth 1A disease) region. Duplication of this region can lead to *PMP22* overexpression and the CMT1A disease phenotype, and deletion of this region can result in insufficient levels of *PMP22* and the HNPP (hereditary neuropathy with liability to pressure palsies)^27^.

Jiaxian (JX), Qinchuan (QC), Nanyang (NY), Jinnan (JN), and Luxi (LX) cattle are five of the eight representative native bovine (*Bos taurus*) breeds in China. They are work and beef dual-purpose breeds and designated as national protection resources due to their excellent performance traits and good meat production characteristics. Chinese Red Steppe (CRS) is a dairy and beef type generated by the cross of shorthorn bulls with Mongolian cows. Some artificial selection and breeding from draft usage to beef production have been applied to this breed for a relatively long time, which might have resulted in different variation. Among isoforms in the CYP4A subfamily, cytochrome P-450 4A11 (CYP4A11) is a major lauric acid (medium-chain fatty acid) omega hydroxylase in human liver and kidney^28^,^29^,^30^. CYP4A11 functions to convert arachidonic acid to 20-hydroxyeicosatetraenoic acid (20-HETE), and is involved in fatty-acid metabolism and blood pressure regulation^30^,^31^. However, there have been no reports about the function of the *CYP4A11* gene in cattle. We previously confirmed that the cattle *CYP4A11* gene was located within CNVR44 that encompasses QTLs that alter economic traits like meat quality and milk production^21^. This location suggests that the copy number of *CYP4A11* must be variable, which we hypothesized may have genetic effects that can be conveyed to phenotypes. This study was performed to confirm the widespread existence of *CYP4A11* CNV in different Chinese cattle breeds, determine its relationship with growth, and explore the biological effects of its expression.

## Results

### Distribution of *CYP4A11* CNV in populations

To validate the presence and investigate the distribution of *CYP4A11* CNV, the relative gene copy number was detected and estimated in six Chinese cattle breeds (Supplementary Table S1). As shown in Table 1, there was a similar distribution of CNV types in JX, QC, NY, JN, LX and CRS populations, with Normal more common than Gain or Loss. Gain represents more than two gene copies, Loss means less than two DNA copies, and Normal means two gene copies. Additionally, there were differences in the distribution frequency among breeds (Table 1).

### Association between *CYP4A11* CNV and growth

Association analysis between *CYP4A11* CNV and growth were carried out for samples from JX, QC, NY, JN, LX, and CRS by one-way analysis of variance (ANOVA). As shown in Table 2, the *CYP4A11* CNV was significantly associated with some growth traits. Specifically, in JX, the adult hucklebone width of Gain type was higher than that of Loss type (*P* < 0.05), while in QC, adult heart girth and chest depth were higher for Gain type than the Loss type (*P* < 0.05); very similarly, NY showed a higher 12-month body length for Gain type than Loss type (*P* < 0.05). Significant effects were also found in JN adults, body weight and heart girth were higher in Gain individuals than in Loss ones (*P* < 0.05). Also, the cannon girth and heart girth in CRS at 6 months were higher in Gain or Normal than in Loss type (*P* < 0.05). Except for the aforementioned results, the other traits were nonsignificantally correlated with *CYP4A11* CNV type but showed a tendency that individuals with Gain or Normal types possessed higher growth values than Loss type (*P* > 0.05), including LX breed (Supplementary Tables S2, S3, S4, S5, S6 and S7).

### Expression differences of *CYP4A11* among different tissues

**Figure 1:**
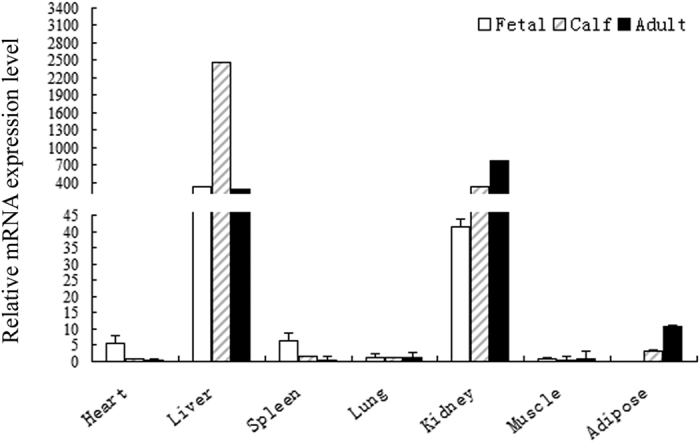
The expression profiling of *CYP4A11* gene at different stages in Qinchuan. Data are presented as means ± standard error (SE, n = 3). Error bars represent standard errors from three independent experiments.

Before analyzing the effect of *CYP4A11* copy number on mRNA level, gene expression profiling was necessary, as the expression pattern of cattle *CYP4A11* had not been examined previously. We determined the expression of *CYP4A11* in seven different tissues in QC from three growth stages (fetal, calf, and adult). The results showed that the *CYP4A11* expression level varied (Fig. 1). In fetal and calf samples, *CYP4A11* mRNA was expressed in all tissues, with a high level in the liver, moderate level in kidney, and low level in the heart, spleen, lung, muscle, and adipose samples. In adults, the mRNA level was the highest in kidney, followed by liver, and the other tissues showed low levels. Additionally, compared with calf, the level of *CYP4A11* expression in the adipose tissue was increased in the adult samples.

### Influence of *CYP4A11* CNV type on mRNA expression

**Figure 2:**
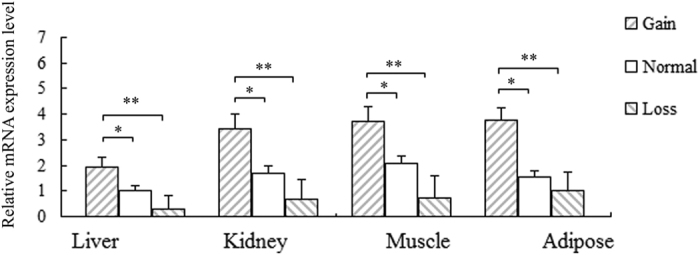
Influence of *CYP4A11* CNV type on gene expression in four types of tissues. Data are presented as means ± standard error (SE, n = 3). Error bars represent standard errors from three independent experiments. Asterisks (*) and (**) indicate statistically significant differences (*P* < 0.05) and (*P* < 0.01), respectively.

Based on the expression profiling, we next analyzed the differences of *CYP4A11* expression level for the different CNV types. As shown in Supplementary Fig. S1, 30 individuals were tested. Among the tested individuals, 21 were of Normal type, 6 were Gain type, and 3 were Loss type. Supplementary Fig. S2 shows the expression in liver, kidney, muscle, and adipose tissues. The CNV type-expression analysis is shown in Fig. 2, which showed that the mRNA level of Gain was significantly higher than that of Normal or Loss type (*P* < 0.01 or *P* < 0.05). No significant difference was found between Normal and Loss, but the mRNA level in Normal was somewhat higher than in Loss. These results indicated that more *CYP4A11* copies increased the gene expression level, and fewer copies decreased gene expression.

### Promotion of overexpressed *CYP4A11* on lipogenesis

**Figure 3:**
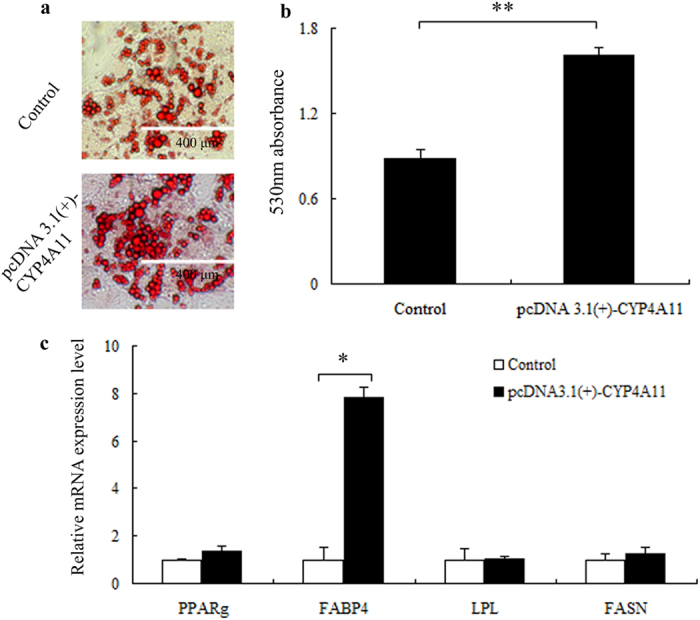
The overexpressed *CYP4A11* in 3T3L1 cells promoted adipogenesis. (**a**) Lipid staining with Oil Red O (ORO). The “Control” indicates the 3T3L1 cell group transfected with pcDNA 3.1(+) plasmids. (**b**) Quantification of ORO staining. Data are presented as means ± standard error (SE, n = 3). Error bars represent standard errors from three independent experiments. “Control” represents the 3T3L1 cell group transfected with pcDNA 3.1(+) plasmids. Asterisks (**) indicate statistically significant differences (*P* < 0.01). (**c**) Comparison of candidate genes expression. Data are presented as means ± standard error (SE, n = 3). Error bars represent standard errors from three independent experiments. “Control” represents the 3T3L1 cell group transfected with pcDNA 3.1(+) plasmids. Asterisks (*) indicate statistically significant differences (*P* < 0.05).

Expression profiling revealed that there was low expression of the *CYP4A11* gene in adipose tissue. To examine the role of *CYP4A11* in fatty-acid metabolism, the protein was overexpressed from a plasmid in 3T3L1 cells. Before differentiation, the mRNA level of *CYP4A11* gene after overexpression was found to be significantly higher than in the control group (*P* = 0.008, Supplementary Fig. S3). The overexpressing cells were exposed to differentiation medium. At the 12^th^ day of induction, Oil Red O (ORO) staining was performed and showed that in the overexpressing cells, there were both larger fat droplets and more droplets than in the cells of the control group (Fig. 3a). The absorbance at 530 nm for the pcDNA 3.1(+)-*CYP4A11* group was 1.606, significantly higher than that of the control group (*P* = 0.001, Fig. 3b). Furthermore, the mRNA level of marker genes important in adipose differentiation, such as peroxisome proliferator-activated receptor gamma (*PPARG*), fatty acid binding protein 4 (*FABP4*), Lipoprotein lipase (*LPL*), and Fatty acid synthase (*FASN*) were 1.34, 7.81, 1.02, and 1.28-fold higher in the overexpression group, respectively, than in the control group, with a significant difference in *FABP4* levels between the two groups (*P* = 0.047, Fig. 3c). These results, together with the previous discovery that additional *CYP4A11* copies increased gene expression level, indicated that *CYP4A11* copy numbers affect preadipocyte differentiation. Based on these results, it could be inferred that the increase in *CYP4A11* copies may promote lipogenesis in cattle.

## Discussion

In livestock such as cattle, most CNV studies have been limited to CNV discovery and enumeration using various platforms, including aCGH, SNP array, and next generation sequencing^20^,^32^,^33^,^34^. Even though these studies have identified many copy number variable regions in different species, there have been few attempts to associate copy number with expression effects. The investigation of the diversity and origin of CNVs, the characterization of their genetic properties, and the determination of the functional impacts of CNVs are promising active areas of research^35^.

The quantitative real-time PCR (qPCR) assay used here was a highly effective method of CNV detection^33^,^36^. Through qPCR analysis, the *CYP4A11* CNV types showed similar distributions in JX, QC, NY, JN, LX, and CRS populations. The Normal type was more common than Gain or Loss types. This indicated the genetic similarity of these breeds after long-term artificial selection and breeding efforts. The six breeds showed differences in CNV frequency that may be caused by the diversity in their genetic backgrounds^37^. JX, QC, NY, JN, and LX cattle are five native Chinese breeds, and CRS is a dairy and beef breed generated by crossing shorthorn bulls with Mongolian cows.

Association analysis revealed that the *CYP4A11* CNVs were significantly associated with some growth traits, however, most of the studied phenotypes showed no significant differences among CNV types, just a tendency of higher growth for Gain or Normal individuals compared to Loss individuals. This reflected the positive effect of multiple copies of *CYP4A11* on growth, which may be attributed to the location of *CYP4A11* in the CNVR44 which includes QTLs that are linked to economic traits^21^. As previous reports showed, some genes were located within CNVRs that directly overlapped with QTLs for body weight, marbling score, and somatic cell count^38^, or were partly encompassed by QTLs for body weight, carcass weight, and breast muscle weight^39^,^40^. *PLA2G2D*, a gene related to milk production and meat quality, was reported to have multiple copies in beef cattle^5^. Consistently, *PLA2G2D* was found to be located in CNVR 22, whose number of repeats can significantly reduce the mRNA levels of *PLA2G2D* and correspondingly cause growth retardation in Chinese cattle^21^. Yue *et al*.^41^,^42^ studied Holstein bulls and found that the *PRAMEY* copy number is negatively correlated with scrotal circumference, relative scrotal circumference, percentage of normal sperm, and nonreturn rate, and the CNVs of both *HSFY* and *ZNF280BY* are negatively correlated with testis size and positively correlated with sire conception rate. This suggests that some phenotypic differences could be attributed to genes encompassed by CNVRs.

CNVs mainly influence phenotypes in three ways: gene dosage, expression regulation changes, and recessive allele exposure. Of these, gene dosage is the major contributor to phenotypes associated with CNVs^43^. Functional genes that are partly encompassed by CNVRs could affect gene transcription or expression to a certain degree^44^,^45^, but genes entirely contained by CNVRs may show a positive relationship with their transcription and expression activity, because the multiple copies could all be actively transcribed and expressed^46^. For instance, the duplication of the *CCL3L1* gene significantly increased its expression level^47^. Similarly, the increase in *CYP4A11* copy numbers increased its expression level, suggesting that the *CYP4A11* CNV may affect cattle phenotypes by altering gene dosage.

To test whether *CYP4A11* CNV functions by gene dosage effects, the *CYP4A11* gene was overexpressed in 3T3-L1 cells. The *CYP4A11* overexpression dramatically increased the formation of fat droplets. At the same time, the expression level of genes related to adipose and lipid accumulation, *PPARG, FABP4, LPL*, and *FASN*, were all increased. *PPARG* is a positive regulator of adipose accretion^48^,^49^. The expression and activity of *LPL* is positively correlated with adipose tissue mass^50^, like *FASN*, which plays a critical role in determining the fatty acid profile^51^. The differences of *PPARG, LPL*, and *FASN* levels between the two groups were not significant. The lack of a dramatic effect may be caused by the strikingly higher *FABP4* level in the pcDNA 3.1(+)-*CYP4A11* group, which increased significantly following lipid accumulation to increase fatty acid degradation, and subsequently reduced the expression levels of *PPARG, LPL*, and *FASN*^52^. These results, together with the finding that the increase in *CYP4A11* copy numbers could increase its expression level, suggest that the *CYP4A11* multicopy may promote 3T3-L1 cell differentiation from preadipocytes to adipocytes and may increase adipose deposits in cattle.

The increase in *CYP4A11* copy numbers promoted cattle growth and increased the expression level, confirming the positive gene dosage effect by which the *CYP4A11* CNV could affect phenotype. This information may be used in future molecular marker-assisted selection for cattle breeding. The *in vitro* overexpression revealed that *CYP4A11* duplication could increase adipose deposits. Future *in vivo* study will provide more insights into the effects of CNV. Taken together, these studies revealed the functional role of the *CYP4A11* CNV and provided the basis for its further exploitation in cattle breeding.

## Methods

### Ethics statement

The experiments and animal care were performed according to the Regulations for the Administration of Affairs Concerning Experimental Animals (Ministry of Science and Technology, China, 2004) and approved by the Institutional Animal Care and Use Committee (College of Animal Science and Technology, Northwest A&F University, China). All the experiments and methods were performed in accordance with the approved guidelines. Cattle were permitted access to feed and water at will and all efforts were made to minimize suffering.

### Animals and samples

Blood samples of JX (N = 246), QC (N = 187), NY (N = 104), JN (N = 102), LX (N = 83), and CRS (N = 66) were respectively collected from Henan, Shannxi, Henan, Shanxi, Shandong, and Jilin provinces. Growth data, including body weight, average daily gain, withers height, body length, heart girth, hucklebone width, hip width, rump length, chest depth, chest breadth, height of hip cross, cannon girth, and abdominal girth were obtained. Tissue samples of fetal (N = 3), calf (N = 3), and adult (N = 3) of QC, including heart, liver, spleen, lung, kidney, muscle and adipose (no adipose for fetal) were collected in a slaughter house. In addition, liver, kidney, muscle, and adipose tissues were obtained from QC adults (N = 30).

### DNA and RNA isolation

Genomic DNA was extracted from blood samples and 30 QC adult livers following standard procedures^53^. Total RNA was isolated from tissues and 3T3-L1 cells by the Trizol method (TaKaRa, Otsu, Shiga, Japan) and reverse-transcribed to get the first-strand cDNA using the Prime Script RT Reagent Kit (TaKaRa, Dalian, China) using 500 ng of total RNA as template and oligo-dT primers. Both the genomic DNA and cDNA were diluted to 20 ng/μL.

### Plasmid construction

The *CYP4A11* coding region was amplified using cDNA as template and inserted into the pcDNA 3.1(+) vector (Invitrogen, Carlsbad, CA). *Nhe*I and *Hind*III restriction sites were included in the forward and reverse primers to amplify the gene. The primers were as follows, sense: 5′-CTAGCTAGCATGAGTGTCTCTGCACTGAGCCCC-3′, and antisense: 5′-CCCAAGCTTCTAAGTCCCTGGATCAGAGAGCTTCCT-3′. The 1563 bp CDS fragments were purified using TIANquick Midi Purification Kit (Tiangen, Beijing, China), then digested with *Nhe*I and *Hind*III. Then, the ligation was carried out overnight at 16 °C and transformed into Top10 *E. coli* cells (Tiangen, Beijing, China). Finally, recombinant plasmid was isolated and confirmed by double digestion and DNA sequencing.

### Cell culture and transfection

3T3-L1 cells (purchased from cellbank.org.cn) were plated into 12-well cell culture dishes and cultured in growth medium comprised of Dulbecco’s Modified Eagle’s Medium (DMEM) (Hyclone, Logan, UT) supplemented with 10% fetal bovine serum (FBS) (HyClone) and 1× antibiotics (100 U/ml penicillin, 100 μg/ml streptomycin; Key-GEN, Nanjing, China) and incubated at 37 °C with 5% CO_2_.

pcDNA3.1(+)-*CYP4A11* and pcDNA3.1(+) vector (control group) were separately transfected into confluent 3T3-L1 cells using Lipofectamine 2000 transfection reagent (Invitrogen, Carlsbad, CA). First, plasmid DNA and Lipofectamine 2000 were diluted in DMEM medium separately at a ratio of 1:2.5. After incubation for 5 minutes at room temperature, the diluted DNA and Lipofectamine 2000 were mixed together and incubated for 20 minutes at room temperature. This complex was then added to a culture dish containing cells and growth medium.

### Cell differentiation and lipid staining

Cell differentiation was induced by addition of differentiation medium. Briefly, six wells of cells were transferred to differentiation medium I (growth medium plus 0.5 mmol/L 3-Isobutyl-1-methylxanthine (IBMX), 1 μmol/L dexamethasone (Dex, Dex medium has a weak ability to induce differentiation of 3T3-L1 cells into adipocytes), and 10 μg/mL Insulin (Promega, Heidelberg, Germany) 24 hours after transfection. Then, about 72 hours later, cells were rinsed twice with phosphate buffered saline (PBS), and differentiation medium I was replaced with differentiation medium II (growth medium plus 10 μg/mL Insulin). Differentiation medium II was renewed every 48 hours until the 12^th^ day when the induction finished. The control group was treated as the same.

Lipid accumulation in adipocytes was visualized by staining with ORO (TaKaRa, Dalian, China). First, three wells of cells were rinsed twice with phosphate buffered saline (PBS) and fixed with 10% formaldehyde for 1 hour at room temperature. Then, after washing twice again with PBS, the cells were stained for at least 20 minutes in freshly diluted ORO solution (0.3% (w/v) ORO in 60% isopropanol). The staining solution was then removed, and cells were washed twice with PBS. ORO was extracted with isopropanol and the signal was quantified spectrophotometrically (NanoPhotometer, Implen Inc., CA, USA) at 530 nm^54^.

### qPCR

To evaluate the CNV type, copy number was estimated by DNA qPCR with the CFX 96TM Real Time Detection System (Bio-Rad, Hercules, CA, USA), using the SYBR Premix Ex Taq II kit (TaKaRa, Dalian, China). Genomic DNA was extracted from blood samples following standard procedures^53^, and was diluted to 20 ng/μL. The primers were designed using Beacon Designer 7 software (Supplementary Table S8), based on the DNA sequence of the *Bos taurus CYP4A11* gene (GenBank accession no. NC_007301.5), with *BTF3* as an internal reference gene^38^. Positive and negative controls were generated, respectively with the genomic DNA of Angus (a beef breed previously used as a reference because it has two DNA copies) and water as templates. Each reaction was performed in triplicate. The 25 μL qPCR amplification volume contained 20 ng genomic DNA, 1 μL of each primer (10 μmol/L), and 12.5 μL SYBR Premix Ex TaqII. The PCR protocol was as follows: 30 s at 95 °C, followed by 40 cycles of 95 °C for 10 s, 60 °C for 20 s, and 68 °C for 20 s.

The differences in *CYP4A11* gene expression in the different tissues were detected by cDNA qPCR. Total RNA was isolated from heart, liver, spleen, lung, kidney, muscle and adipose tissues by the Trizol method (TaKaRa, Otsu, Shiga, Japan). The quantity and quality of the RNA were determined using a NanoPhotometer spectrometer (ImplenInc, CA, USA). Next, the RNA was reverse-transcribed to obtain the first-strand cDNA using the Prime Script RT Reagent Kit (TaKaRa, Dalian, China) using 500 ng of total RNA as template and oligo-dT primers. After synthesis, the cDNA template was diluted to 20 ng/μL. As a housekeeping gene, glyceraldehyde-3-phosphate dehydrogenase (*GAPDH*) was selected for the endogenous standard^55^. The specific primers for *CYP4A11* and *GAPDH* gene are listed in Supplementary Table S8. The qPCR amplification volume and protocol was performed the same as the genomic DNA qPCR. Every reaction was performed in triplicate.

To evaluate the influences of *CYP4A11* copy numbers on expression level, genomic DNA and total RNA samples were isolated from 30 QC individuals and qPCR was performed. Genomic DNA was isolated from livers^53^ and total RNA was isolated from liver, kidney, muscle, and adipose tissues and reversed-transcribed. The genomic DNA qPCR and cDNA qPCR amplification were respectively performed following methods described above. Every reaction was performed in triplicate.

To assess the effects of multiple *CYP4A11* copies on adipogenesis, the expression levels of *CYP4A11, PPARG, FABP4, LPL*, and *FASN* in 3T3-L1 cells were determined for the two groups (pcDNA3.1(+)-*CYP4A11* group and control group) by qPCR. After transfection, RNA was isolated from three wells of cells and the synthesized cDNA was used to detect the *CYP4A11* expression level. After differentiation, three wells of cells were gathered for RNA isolation and subsequent expression level detection of *PPARG, FABP4, LPL*, and *FASN* genes. The primer sequences for the tested genes and the internal control gene *GAPDH* are listed in Supplementary Table S9. RNA isolation, cDNA synthesis, and qPCR amplification were performed as described above. Every reaction was performed in triplicate.

### CNV type assay

The qPCR analysis was performed based on the ΔΔCt method^56^, and ΔΔCt values were determined by comparing tested samples and the Angus reference, using the *BTF3* gene as an internal control. Finally, the relative copy number for each sample was calculated as 2^−ΔΔCt^. By comparing the 2^−ΔΔCt^ value of the tested individuals with the value for Angus, the CNV type of the *CYP4A11* gene was defined as Gain (more than two gene copies), Loss (less than two gene copies), or Normal (two gene copies, same as the Angus reference).

### Association analysis

Association analysis between the CNV types of *CYP4A11* and growth traits was performed using the General Linear Model (GLM) in SPSS (version 16.0).

The linear model was: *Y*_*ijk*_ = *μ* + *A*_*i*_ + *T*_*j*_ + *e*_*ijk*_, where *Y*_*ijk*_ is the phenotypic value of the *ijk*th individual, *μ* is the overall population mean, *A*_*i*_ is the effect of the *i*th age, *T*_*j*_ is the effect associated with the *j*th CNV type, and *e*_*ijk*_ is the random error.

### Expression analysis

The relative expression levels of *CYP4A11, PPARG, FABP4, LPL*, and *FASN* were calculated using Gene Expression Macro software (Applied Biosystems, Life Technologies, Carlsbad, CA, USA) by normalizing to the expression of *GAPDH* with the ΔΔCt method^56^.

The differences of mRNA expression level among three *CYP4A11* CNV types were estimated by ANOVA. The results of the multiple comparisons were corrected by Bonferroni correction, and the differences were considered significant if *P* < 0.05.

The differences of expression levels in *CYP4A11, PPARG, FABP4, LPL*, and *FASN* genes between pcDNA3.1(+)-*CYP4A11* and the control group were evaluated by the Student’s t-test (two-tail). The differences were considered significant if *P* < 0.05.

## Additional Information

**How to cite this article:** Yang, M. J. *et al*. Association study and expression analysis of *CYP4A11* gene copy number variation in Chinese cattle. *Sci. Rep.* **7**, 46599; doi: 10.1038/srep46599 (2017).

**Publisher's note:** Springer Nature remains neutral with regard to jurisdictional claims in published maps and institutional affiliations.

## Supplementary Material

Supplementary Dataset 1

Supplementary Dataset 2

## Figures and Tables

**Table 1:** Distribution of the *CYP4A11* gene CNV types among breeds.

Breeds	Sample size	CNV type		
		Gain	Normal	Loss
Jiaxian	246	40 (16.26%)	187 (76.02%)	19 (7.72%)
Qinchuan	187	34 (18.18%)	133 (71.12%)	20 (10.70%)
Nanyang	104	32 (30.77%)	61 (58.65%)	11 (10.58%)
Jinnan	102	4 (3.92%)	77 (75.49%)	21 (20.59%)
Luxi	83	19 (22.89%)	44 (53.01%)	20 (24.10%)
Chinese Red Steppe	66	19 (28.79%)	41 (62.12%)	6 (9.09%)

**Table 2:** Significant associations of *CYP4A11* CNV types with growth traits.

Breeds	Growth traits	CNV Type		
		Gain (Mean ± SE)	Normal (Mean ± SE)	Loss (Mean ± SE)
Jiaxian	Adult HW (cm)	26.76^a^ ± 0.54	26.08^a,b^ ± 0.35	24.30^b^ ± 1.05
Qinchuan	Adult HG (cm)	182.39^a^ ± 1.83	177.67^a,b^ ± 7.61	174.90^b^ ± 2.95
	Adult CD (cm)	65.06^a^ ± 0.97	62.33^a,b^ ± 4.06	61.05^b^ ± 1.57
Nanyang	12 months BL (cm)	119.14^a^ ± 2.59	116.55^a,b^ ± 1.11	111.17^b^ ± 2.80
Jinnan	Adult BW (kg)	489.02^a^ ± 10.82	488.25^a^ ± 45.91	437.15^b^ ± 21.07
	Adult HG (cm)	186.01^a^ ± 1.48	183.25^a,b^ ± 6.29	179.53^b^ ± 2.89
Chinese Red Steppe	6 months HG (cm)	133.92^a^ ± 2.81	133.62^a^ ± 1.47	127.70^b^ ± 1.84
	6months CG (cm)	12.58^a^ ± 0.15	12.47^a^ ± 0.12	11.83^b^ ± 0.23

Values with different superscripts within the same line differ significantly at *P* < 0.05 ^a,b^SE: standard error. HW, Hucklebone width; HG, heart girth; CD, Chest depth; BL, body length; BW, body weight; CG, Cannon girth.
