# Supplementary material for: Association study and expression analysis of CYP4A11 gene copy number variation in Chinese cattle
Source: Sci Rep. 2017 May 11;7:46599. doi: 10.1038/srep46599 (PMC5425913; doi:10.1038/srep46599)

Association study and expression analysis of CYP4A11 gene copy number variation in Chinese cattle

M. J. Yang[1,2], J. Q. Lv[3], L. Z. Zhang[1], M. X. Li[1], Y. Zhou[1], X. Y. Lan[1], C. Z. Lei[1] & H. Chen[1*]

Supplementary Table S1. The distribution of *CYP4A11* CNV types in Jiaxian, Qinchuan, Nanyang, Jinnan, Luxi and Chinese Red Steppe breed. (XLS)

Supplementary Table S2. Nonsignificant associations of *CYP4A11* CNV types with growth traits in Jiaxian adults.

Supplementary Table S3. Nonsignificant associations of *CYP4A11* CNV types with growth traits in Qinchuan adults.

Supplementary Table S4. Nonsignificant associations of *CYP4A11* CNV types with growth traits in Nanyang breed.

Supplementary Table S5. Nonsignificant associations of *CYP4A11* CNV types with growth traits in Jinnan adults.

Supplementary Table S6. Nonsignificant associations of *CYP4A11* CNV types with growth traits in Luxi adults.

Supplementary Table S7. Nonsignificant associations of *CYP4A11* CNV types with growth traits in Chinese Red Steppe breed.

Supplementary Table S8. Primers used for CNV detection and expression profiling of *CYP4A11* gene.

Supplementary Table S9. Primer information of the candidate genes in *CYP4A11* overexpression study.

Supplementary Figure S1. Distribution of the *CYP4A11* CNV types in Qinchuan.

Supplementary Figure S2. Relative expression level of *CYP4A11* in four tissues in Qinchuan.

Supplementary Figure S3. Overexpression efficiency of *CYP4A11* in 3T3L1 cells.

Supplementary Table S2. Nonsignificant associations of *CYP4A11* CNV types with growth traits in Jiaxian adults. SE: standard error.

Growth traits	CNV Type		
	Gain (Mean ±SE)	Normal (Mean ±SE)	Loss (Mean ±SE)
Body weight (kg)	350.44 ±21.01	347.63 ±6.93	340.11 ±10.78
Withers height (cm)	124.00 ±2.17	122.32 ±0.72	121.84 ±1.12
Body length (cm)	138.30 ±2.89	135.60 ±0.95	134.42 ±1.48
Heart girth (cm)	165.60 ±1.18	165.03 ±1.84	165.40 ±3.59
Hip width (cm)	43.32 ±0.72	43.14 ±0.47	41.90 ±1.41
Rump length (cm)	43.53 ±0.52	43.08 ±0.33	43.90 ±1.01
Chest depth (cm)	63.20 ±1.40	60.88 ±0.46	60.58 ±0.72
Chest breadth (cm)	38.40 ±1.14	36.76 ±0.59	36.59 ±0.38
Height of hip cross (cm)	129.40 ±2.44	126.44 ±0.80	126.58 ±1.25

Supplementary Table S3. Nonsignificant associations of *CYP4A11* CNV types with growth traits in Qinchuan adults. SE: standard error.

Growth traits	CNV Type		
	Gain (Mean ±SE)	Normal (Mean ±SE)	Loss (Mean ±SE)
Body weight (kg)	431.64 ±11.31	422.08 ±47.09	392.20 ±18.24
Withers height (cm)	130.33 ±3.83	130.08 ±0.92	128.95 ±1.48
Body length (cm)	144.00 ±5.95	138.35 ±1.43	137.90 ±2.30
Hucklebone width (cm)	23.69 ±0.50	22.70 ±0.80	23.33 ±2.07
Hip width (cm)	43.40 ±0.55	42.10 ±0.88	41.33 ±2.28
Rump length (cm)	46.67 ±1.93	44.11 ±0.47	42.60 ±0.75
Chest breadth (cm)	38.46 ±0.67	37.33 ±1.04	33.67 ±1.04
Height of hip cross (cm)	129.13 ±2.18	127.41 ±1.35	127.67 ±5.64

Supplementary Table S4. Nonsignificant associations of *CYP4A11* CNV types with growth traits in Nanyang breed. SE: standard error.

Age	Growth traits	CNV Type		
		Gain (Mean ±SE)	Normal (Mean ±SE)	Loss (Mean ±SE)
Birth	Birth weight (kg)	30.50 ±1.00	29.55 ±0.40	29.50 ±0.93
Six months	Body weight (kg)	161.43 ±7.05	157.55 ±3.03	154.17 ±7.61
	Average daily gain (kg)	0.73 ±0.04	0.71 ±0.02	0.69 ±0.04
	Body height (cm)	106.29 ±1.78	105.95 ±0.76	102.00 ±1.92
	Body length (cm)	105.71 ±2.03	105.40 ±0.87	103.00 ±2.19
	Heart girth (cm)	129.43 ±2.24	127.82 ±0.96	126.17 ±2.42
	Hucklebone width (cm)	18.71 ±0.51	18.43 ±0.22	18.58 ±0.55
Twelve months	Body weight (kg)	228.29 ±8.60	220.55 ±3.69	209.67 ±9.29
	Average daily gain (kg)	0.37 ±0.05	0.35 ±0.02	0.31 ±0.05
	Body height (cm)	114.14 ±1.47	113.84 ±0.63	112.33 ±1.58
	Heart girth (cm)	142.00 ±2.56	140.21 ±1.10	139.17 ±2.76
	Hucklebone width (cm)	21.14 ±0.54	20.82 ±0.23	20.75 ±0.59
Eighteen months	Body weight (kg)	307.29 ±12.12	300.08 ±5.20	297.83 ±13.09
	Average daily gain (kg)	0.49 ±0.08	0.44 ±0.08	0.44 ±0.03
	Body height (cm)	121.57 ±3.25	121.92 ±1.40	121.33 ±3.51
	Body length (cm)	129.57 ±2.59	128.42 ±1.11	127.17 ±2.80
	Heart girth (cm)	156.18 ±1.42	155.14 ±3.23	155.33 ±3.56
	Hucklebone width (cm)	23.42 ±0.71	23.13 ±0.28	22.86 ±0.66
Twenty-four months	Body weight (kg)	381.00 ±17.06	371.45 ±7.32	359.33 ±18.42
	Average daily gain (kg)	0.41 ±0.06	0.40 ±0.02	0.34 ±0.06
	Body height (cm)	127.29 ±1.81	127.00 ±1.96	126.37 ±0.78
	Body length (cm)	139.00 ±2.87	137.40 ±1.23	135.50 ±3.10
	Heart girth (cm)	169.16 ±1.53	167.43 ±3.57	166.25 ±3.86
	Hucklebone width (cm)	25.29 ±0.90	25.25 ±0.39	25.17 ±0.97

Supplementary Table S5. Nonsignificant associations of *CYP4A11* CNV types with growth traits in Jinnan adults. SE: standard error.

Growth traits	CNV Type		
	Gain (Mean ±SE)	Normal (Mean ±SE)	Loss (Mean ±SE)
Withers height (cm)	127.42 ±0.60	126.50 ±2.55	127.21 ±1.17
Body length (cm)	156.50 ±4.88	153.76 ±1.15	151.63 ±2.24
Height of hip cross (cm)	128.50 ±2.73	128.84 ±1.25	128.35 ±0.64

Supplementary Table S6. Nonsignificant associations of *CYP4A11* CNV types with growth traits in Luxi adults. SE: standard error.

Growth traits	CNV Type		
	Gain (Mean ±SE)	Normal (Mean ±SE)	Loss (Mean ±SE)
Body weight (kg)	442.27 ±20.94	404.80 ±36.26	388.50 ±40.54
Withers height (cm)	134.20 ±2.31	133.20 ±1.33	133.75 ±2.58
Body length (cm)	149.25 ±6.64	149.33 ±3.43	149.20 ±5.94
Heart girth (cm)	188.73 ±2.65	184.60 ±4.59	181.75 ±5.14
Cannon girth (cm)	18.40 ±0.15	18.25 ±0.29	18.20 ±0.26
Abdominal girth (cm)	216.33 ±4.18	207.00 ±8.09	201.00 ±8.09

Supplementary Table S7. Nonsignificant associations of *CYP4A11* CNV types with growth traits in Chinese Red Steppe breed. SE: standard error.

Age	Growth traits	CNV Type		
		Gain (Mean ±SE)	Normal (Mean ±SE)	Loss (Mean ±SE)
Six months	Body weight (kg)	152.13±3.85	148.94±7.37	139.75±4.83
	Withers height (cm)	104.67±1.60	102.72±1.27	100.44±2.44
	Body length (cm)	107.02±1.19	106.27±0.95	103.82±1.83
Twenty-four months	Body weight (kg)	501.40±17.58	486.44±46.52	482.18±32.89
	Withers height (cm)	129.21±1.80	128.30±0.68	128.06±1.27
	Body length (cm)	127.94±0.93	127.70±0.50	127.58±1.32
	Heart girth (cm)	214.50±3.68	211.62±9.74	210.17±6.88
	Cannon girth (cm)	15.09±0.08	14.96±0.14	14.93±0.20

Supplementary Table S8. Primers used for CNV detection and expression profiling of *CYP4A11* gene.

Gene name	Primer sequences	Product length
CYP4A11	F: 5'- CCTGGTTCTGCTTCTGAT -3'<br>R: 5'- AAGTGATGAAGGGCTTTGA -3'	75 bp
BTF3	F: 5'- CAAGAAGACTCATTCCTT -3'<br>R: 5'- CACAAGCACATTATTCAC -3'	109 bp
GAPDH	F: 5'- TGAGGACCAGGTTGTCTCCTGCG -3'<br>R: 5'- CACCACCCTGTTGCTGTAGCCA -3'	145 bp

Supplementary Table S9. Primer information of the candidate genes in *CYP4A11* overexpression study.

Candidate gene	Primer sequences	Reference sequence	Product length
GAPDH	F: 5'- AGTGGCAAAGTGGAGATT -3'<br>R: 5'- GTGGAGTCATACTGGAACA -3'	NM_001289726	83 bp
PPARg	F: 5'- GCATCAGGCTTCCACTAT -3'<br>R: 5'- CTTCAATCGGATGGTTCTTC -3'	NM_001127330	75 bp
FABP4	F: 5'- TGGTGGAATGTGTTATGAA -3'<br>R: 5'- TGCTTGCTTATTAGTGGAA -3'	NM_024406	160 bp
LPL	F: 5'- AGTCTGTTGTGGTTATCTG -3'<br>R: 5'- GTTAAGTTGGCTCAGTGA -3'	NM_008509	92 bp
FASN	F: 5'- AATAGCATCATCCTCTACTTG -3'<br>R: 5'- CTCACAGTGGTCACATAC -3'	NM_007988	86 bp

Supplementary Figure S1. Distribution of the *CYP4A11* CNV types in Qinchuan (n = 30).

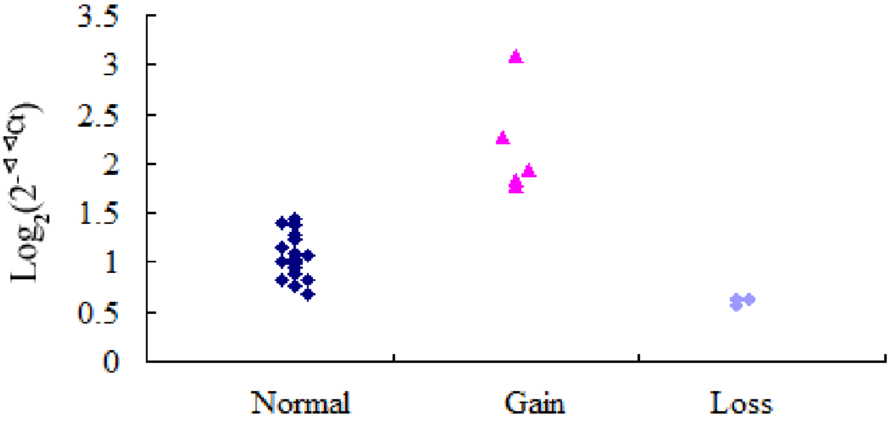

Supplementary Figure S2. Relative expression level of *CYP4A11* in four tissues in Qinchuan (n = 30). (a) liver. (b) kidney. (c) muscle. (d) adipose. Error bars represent standard errors from three independent experiments.

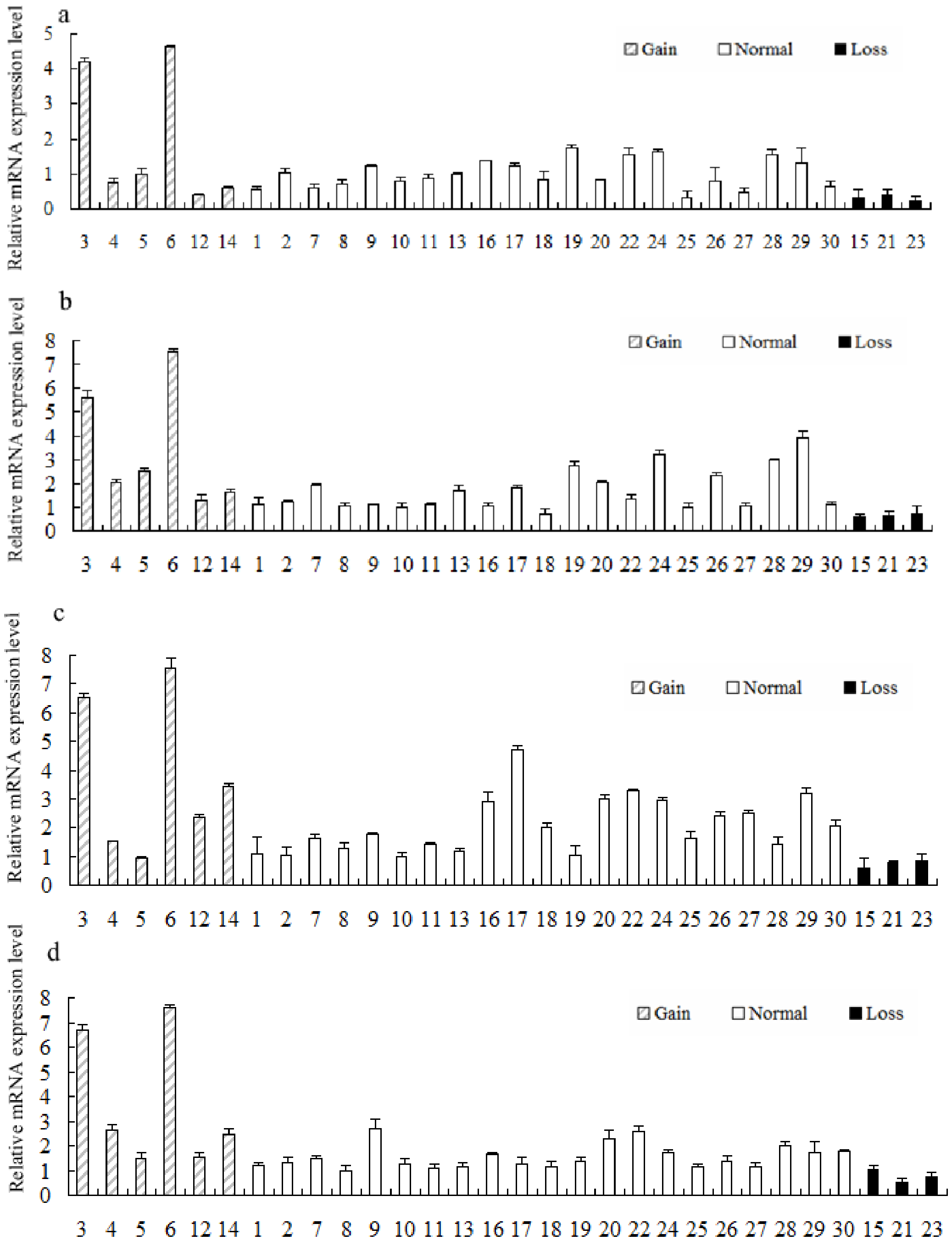

Supplementary Figure S3. Overexpression efficiency of *CYP4A11* in 3T3L1 cells. Data were presented as means ± standard error (SE, n=3). Error bars represent standard errors from three independent experiments. "Control" presented the 3T3L1 cell group transfected with pcDNA 3.1(+) plasmids. Asterisks (**) indicate statistically significant differences (*P*<0.01).

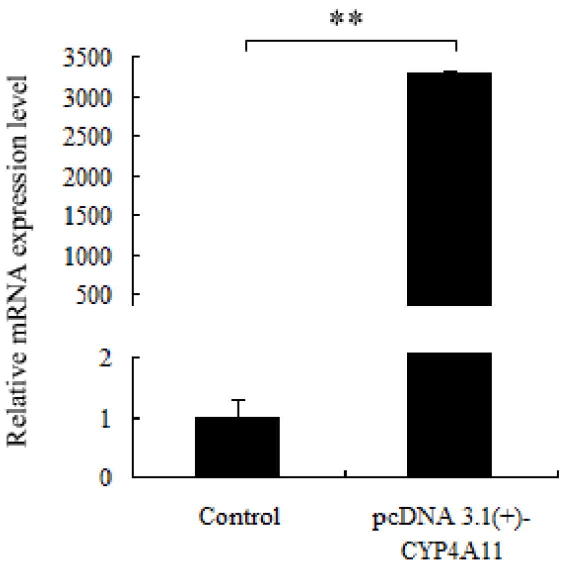

Supplement: Supplementary Dataset 2 [file srep46599-s2.pdf]
